# Efficacy and safety of different doses of tenecteplase for the treatment of acute ischemic stroke: A protocol for a systematic review and network meta-analysis

**DOI:** 10.1097/MD.0000000000023379

**Published:** 2020-12-04

**Authors:** Ting Shen, Jinjian Zhou, Yan Zhao

**Affiliations:** a Department of Neurology, Zhuji Hospital Affiliated to Shaoxing University of Arts and Science, Zhuji, Shaoxing; b Department of Neurology, People's Hospital of Banan District of Chongqing, Chongqing, China.

**Keywords:** intravenous thrombolysis, ischemic stroke, network meta-analysis, protocol, systematic review, tenecteplase

## Abstract

**Background:**

Acute ischemic stroke (AIS) has become the major reason of causing death around the world. As a newer generation fibrinolytic agent, the potential of tenecteplase in treating AIS has been determined in clinical studies and meta-analysis. However, various doses have been prescribed for tenecteplase in clinical practice, and the optimal dose is not yet clear.

**Methods:**

We will perform a systematic search to capture all potential randomized controlled trials (RCTs) of persons with confirmed AIS who were instructed to administer tenecteplase that report at least one outcome in PubMed, Embase, and the Cochrane Library. Two reviewers will independently check the titles, abstracts, and full-texts, extracting data, assessing the risk of bias and evaluating the certainty of evidence. We will use a random-effect model based on the Bayesian framework to completely direct and network meta-analyses. We will also test the robustness of all pooled results through conducting subgroup analyses according to the following criteria:

1. low and high risk of bias;
2. impact factors (≥5, 3–5, and ≤3);
3. usage of endovascular thrombectomy or not.

**Discussion:**

Our systematic review and network meta-analysis will generate several valuable findings and have several strengths including:

1. a systematic search string;
2. predesigned criteria for subgroup analysis;
3. determination of level of evidence with the GRADE system; and
4. graphic illustration of numerical results.

We therefore believe that findings from this network meta-analysis will benefit future study design and improve evidence-based treatment of AIS.

**Ethics and dissemination:**

We will disseminate the results from the present study through submitting it to conferences or peer-reviewed journal.

**Protocol registry:**

The protocol of our systematic review and network meta-analysis was registered in International Plateform of Registered Systematic Review and Meta-Analysis Protocols (INPLASY) platform with an approval number of INPLASY2020100086 (https://inplasy.com/inplasy-2020-10-0086/). Moreover, this protocol was funded through a protocol registry.

## 1 Introduction

Acute ischemic stroke (AIS), which is a clinical result resulted from cerebrovascular insufficiency due to cerebrovascular stenosis or occlusion, has become the one of the major contributors to deaths worldwide.^[1]^ Early thrombolytic therapy has been regarded as the effective treatment for AIS.^[2]^ As one option of thrombolytic therapy, tenecteplase which is a genetically modified variant of alteplase, has been used to treat AIS in clinical practice.^[3]^

Up to date, several published studies reported the efficacy and safety of tenecteplase in treating AIS, and suggested that tenecteplase may be a potential agent for effectively treating AIS.^[4–6]^ Moreover, meta-analyses which investigated the comparative efficacy and safety of tenecteplase vs alteplase also determined the value of tenecteplase for the treatment of AIS.^[3,7,8]^ However, practitioners prescribed various doses when they considered tenecteplase to treat AIS, and no study to further determine the optimal dose of tenecteplase currently in clinical studies.^[9]^ Therefore, it is imperative to design new study to answer this question.

Considering the drawback that traditional head-to-head meta-analysis cannot simultaneously investigate the comparative efficacies of more than two interventions, network meta-analysis has been developed and then has been extensively used to simultaneously explore the comparative efficacies of three or above.^[8,10]^ So, we designed this study with network meta-analysis technique to investigate the comparative efficacy and safety of different doses of tenecteplase in treating AIS for the purpose of determining the optimal prescribed dose of tenecteplase in clinical practice.

## 2 Methods

### 2.1 Study registration

We designed the present protocol of systematic review and network meta-analysis according to recommendations proposed by the Cochrane Collaboration.^[11]^ We reported this protocol in accordance with the preferred reporting items for systematic review and meta-analysis protocols (PRISMA-P) 2015 statement.^[12]^ We also followed the PRISMA Extension Statement for Reporting of Systematic Reviews Incorporating Network Meta-Analyses of Health Care Interventions.^[13]^ Our protocol was funded through a protocol registry because we registered this protocol on the International Plateform of Registered Systematic Review and Meta-Analysis Protocols (INPLASY) website and received an unique register number of INPLASY2020100086 (accessing at: https://inplasy.com/inplasy-2020-10-0086/).^[14]^ Ethical approval and informed consent were not required because all statistical analyses in this systematic review and network meta-analysis would be conducted based on previous data.

### 2.2 Eligibility criteria

We designed the following inclusion criteria according to our aims:

1. patients confirmed with acute cerebral ischemia;
2. tenecteplase vs other active comparators such as alteplas; and
3. randomized clinical trial.

A study would be excluded if at least one of the following criteria would be covered:

1. essential data for the final analysis is not accessible;
2. duplicate studies with insufficient data or poor quality;
3. ineligible study design such as review, case report, or experimental study.

### 2.3 Definition of outcomes

We defined disability-free outcome, which is evaluated to have a score between 0 and 1 at the modified Rankin Scale [mRS], at 3 months poststroke as the primary outcome of interesting. Functional independence (mRS, 0–2) at 3 months, reduced level of disability overall 7 mRS levels at 3 months, symptomatic intracranial hemorrhage (sICH), and mortality was defined as secondary outcomes of interesting. Symptomatic hemorrhage events were identified using the sICH definition employed in individual trial.^[3]^ Two independent investigators will extract data. Any disagreements would be resolved through consulting a third senior investigator.

### 2.4 Identification and selection of studies

We will perform a systematic search in PubMed, Embase, and the Cochrane Library to capture any potentially eligible records. No publication status and language would be imposed. We will use the combination of MeSh and text to construct the search string and update the details of strings according to the unique requirements of each database. As an example, we document the search string of PubMed in supplementary material. We will also check the reference lists of all eligible studies and other pertinent publications to identify any relevant study. The final search would be ended until the final version of the manuscript was prepared. Two independent investigators will perform the search, and any divergences of identification of studies would be resolved through consulting a third senior investigator.

**Figure 1:**
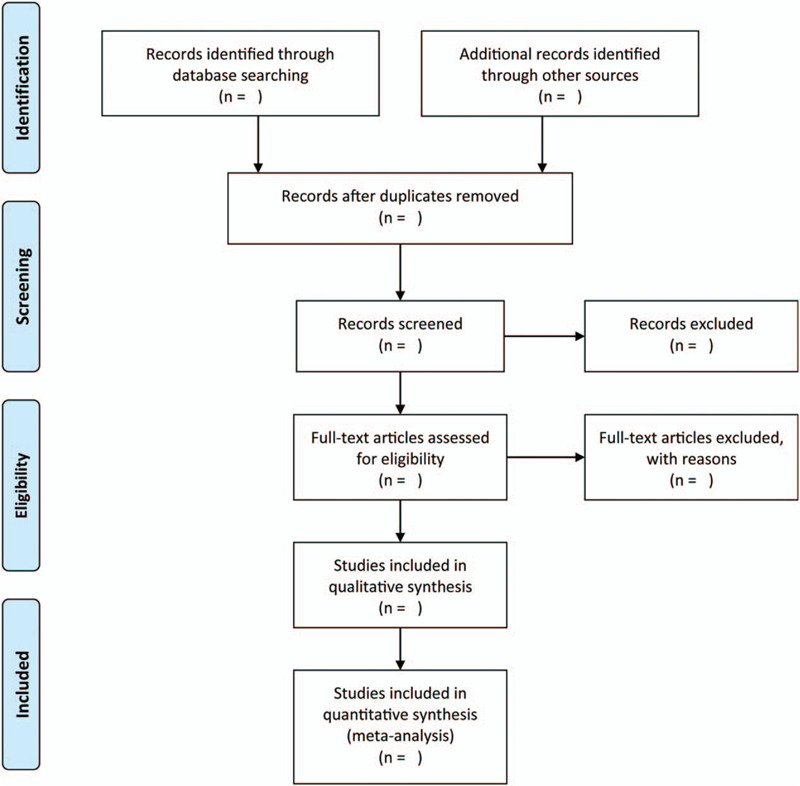
The flow diagram of identification and selection of eligible studies.

Two independent investigators will be assigned to check the eligibility of each study based on EndNote software. Disagreements will be resolved between these two investigators about study selection through consensus principle. Reasons for excluding any studies will be recorded, and a PRIMSA flow diagram (Fig. 1) will be used to display the process of identification and screening of studies.

### 2.5 Data extraction

We will predesign a standardized data extraction sheet based on Microsoft Excel software to extract essential data. The following items including basic information of study, basic information of participants, clinical characteristics of participants, details of regimes, details of the risk of bias, and outcomes of interesting. Leading author will be contacted when essential data were missed. We will estimate mean and standard deviation (SD) for a continuous outcome from the median, range, and the size of the study sample when not available to be extracted.^[15]^ Disagreements will be resolved between the investigators will be resolved through a consensus principle.

### 2.6 Assessment of risk of bias

Two investigators will independently assess the risk of bias of each eligible study with the Cochrane risk of bias tool as the following domains: selection bias (random sequence generation and allocation concealment), performance bias, detection bias, attrition bias, reporting bias, and other sources of bias.^[16]^ According to the matched level of actual information and assessment criteria, a study will be rated as low, unclear, or high risk of bias for a given domain.^[11]^ Any divergences about the assessment of risk of bias will be resolved through consensus principle between two investigators.

### 2.7 Statistical analysis

In our meta-analysis, we will use the odds ratio (OR) with corresponding 95% credible intervals (CrIs) and MDs with corresponding SDs to express the summary effect of dichotomous outcomes and continuous outcomes, respectively. We will qualitatively check the heterogeneity among eligible studies with the Q statistic, and then quantitatively estimated the level of heterogeneity using *I*^2^ statistic.^[17]^ Studies will be considered for homogenesis if *P* > .10 and *I*^2^ < 50%, or studies will be considered to be heterogeneous.^[11]^ Moreover, we used net changes in measurements, which were estimated using the method recommended by Cochrane handbook^[18]^ to estimate the efficacy of all treatments. The result will be considered to be statistically significant when *P* value <.05. We will perform direct meta-analysis based on the random-effect model with Review Manager (RevMan) version 5.3 (Cochrane Collaboration, Copenhagen, Denmark).

We will also conduct network meta-analysis to explore the comparative efficacy of various doses of tenecteplase using OpenBUGS software version 3.2.3 (MRC Biostatistics Unit, Cambridge, UK) following the methods proposed by Lu and Ades.^[10,19]^ We will use the initial value which is automatically generated from software to fit the model.^[20]^ We will perform each Markov chain Monte Carlo chain with 70,000 iterations and 30,000 burn-in in order to obtain satisfactory convergence.

### 2.8 Subgroup analysis

We will test the robustness of all pooled results through conducting subgroup analyses according to the following criteria:

1. low and high risk of bias;
2. impact factors (≥5, 3–5, and ≤3);
3. usage of endovascular thrombectomy or not.

### 2.9 Network geometry and presentation of results

We will qualitatively describe the evidence network of various doses and graphically display the features of the network. In this evidence network, the size of individual node and the width of individual edge is corresponding to the amount of information they contain.^[21]^ The larger nodes and wider edges will be considered to contribute more to the network meta-analysis.^[21]^ We will document all pooled estimates and corresponding 95% CrIs from network meta-analysis using the league table and forest plot. We will illustrate the probabilities that a certain regime become the optimal option with the cumulative probability rankograms.^[22,23]^ We will also calculate the surface under the cumulative ranking (SUCRA) curves to exhibit the hierarchy of doses.

### 2.10 Assessment of quality of evidence

We will assess the quality of evidence according to the calibration exercises for direct and indirect evidence with the Grading of Recommendations Assessment, Development and Evaluation (GRADE) system.^[24]^ The level of direct evidence will be labeled as very low, low, moderate, or high according to 5 aspects including risk of bias, inconsistency, indirectness, imprecision, and publication bias.^[24]^ The level of indirect evidence will be assessed according to the same criteria with additional consideration for intransitivity. The first order loop comparisons of two direct comparisons will be considered to the base for assessing the level of the district comparison because the contribution of the first loop to the indirect evidence is generally the greatest and most precise. The lower level of the two direct comparison will be used to rate the level of indirect evidence and may be decreased when the transitivity was not identified. If a comparison was only obtained from indirect evidence, it is essential to critically assess the incoherence and intransitivity. If a comparison was generated from both direct and indirect evidence, the higher level of the two types of evidence will be used to rate the level of evidence based on network meta-analysis.

### 2.11 Publication bias

We will draw the adjusted funnel plot to qualitatively inspect whether the presence of publication bias when accumulated numbers of eligible studies for individual outcome of interesting was more than 10, and we will further conduct Egger test to test the symmetry of funnel plot or not in order to quantitatively test whether the presence of publication bias.^[25,26]^

## 3 Discussion

### 3.1 Rational basis of performing meta-analysis

AIS is a cerebral infarction resulted from cerebral artery occlusion, which also accompanied by damage of neurons, astrocytes, and oligodendrocytes. AIS has been the leading one of vascular reason in central never system of causing disability and even death worldwide.^[1]^ As a genetically modified variant of alteplase, tenecteplase has been used to treat AIS and several randomized controlled trials and meta-analyses has also been suggested tenecteplase was not inferior to alteplase.^[3,7]^ However, different doses of tenecteplase were used in clinical practice and lack of head-to-head study evaluating the relative efficacy of different doses of tenecteplase used in the treatment of AIS,^[14]^ warrants the completion of a methodologically sound systematic review and, if possible, network meta-analysis to help better inform AIS treatment recommendations.

### 3.2 The importance of main findings

We designed the current systematic review and network meta-analysis to first explore the efficacy and safety of different does of tenecteplase in treating AIS, which will be associated with the following several methodological strengths:

1. a systematic search strategy;
2. two independent investigators completed the screening and data extraction;
3. performing subgroup analysis according to the predesigned criteria;
4. application of GRADE to assess the level of evidence.

After completed the current systematic review and network meta-analysis, we will obtain more reliable and robust findings about tenecteplase for AIS, which will better inform health care professionals and policy makers resulting in improved evidence-based clinical management. Moreover, currently understudied doses comparisons may be detected by evaluation of the network to guide future research.

### 3.3 Limitations of this network meta-analysis

Although we will obtain several strengths in this network meta-analysis, many limitations could not be ignored, such as variability in included randomized controlled trial methods, considerable unexplained heterogeneity, uncertainty in effect estimates, and unbalanced or underpowered networks. This review hopes to mediate some of these potential challenges through its comprehensiveness, a priori specified exploration of heterogeneity and sensitivity analyses, whenever possible.

### 3.4 Ethics and dissemination

We do not need ethics approval and informed consent in the current systematic review and meta-analysis because we will perform all statistical analyses based on published data. After completed this systematic review and meta-analysis, we will submit it to be considered for publication in a peer-reviewed scholarly journal. Moreover, we will also submit our findings to gain more communications in some important conferences.

## Acknowledgments

We will appreciate the International Plateform of Registered Systematic Review and Meta-Analysis Protocols (INPLASY) platform to accept our application of registering the current protocol.

## Author contributions

TS, JJZ, and YZ conceived and designed the current protocol. TS and YZ reviewed scoping searches and contributed to the methodological development of the protocol. TS drafted the manuscript and other authors (JJZ and YZ) critically made a revision. All authors reviewed and approved the final version for publication. YZ is the review guarantor.

**Conceptualization:** Ting Shen, Jinjian Zhou, Yan Zhao.

**Investigation:** Ting Shen, Yan Zhao.

**Methodology:** Ting Shen, Yan Zhao.

**Supervision:** Yan Zhao.

**Validation:** Yan Zhao.

**Writing – original draft:** Ting Shen.

**Writing – review & editing:** Jinjian Zhou, Yan Zhao.

## Supplementary Material

Supplemental Digital Content
