# Supplementary material for: Efficacy and safety of different doses of tenecteplase for the treatment of acute ischemic stroke: A protocol for a systematic review and network meta-analysis
Source: Medicine (Baltimore). 2020 Dec 4;99(49):e23379. doi: 10.1097/MD.0000000000023379 (PMC7717732; doi:10.1097/MD.0000000000023379)
Supplement: Supplemental Digital Content [file medi-99-e23379-s001.docx]

**PubMed search strategy**

**(((("Stroke"[Mesh]) OR ((((((((((((((((((stroke[Title/Abstract]) OR (Strokes[Title/Abstract])) OR (Cerebrovascular Accident[Title/Abstract])) OR (Cerebrovascular Accidents[Title/Abstract])) OR (CVA[Title/Abstract])) OR (CVAs[Title/Abstract])) OR (Cerebrovascular Apoplexy[Title/Abstract])) OR (Brain Vascular Accident[Title/Abstract])) OR (Brain Vascular Accidents[Title/Abstract])) OR (Cerebrovascular Stroke[Title/Abstract])) OR (Cerebrovascular Strokes[Title/Abstract])) OR (Apoplexy[Title/Abstract])) OR (Cerebral Stroke[Title/Abstract])) OR (Cerebral Strokes[Title/Abstract])) OR (Acute Stroke[Title/Abstract])) OR (Acute Strokes[Title/Abstract])) OR (Acute Cerebrovascular Accident[Title/Abstract])) OR (Acute Cerebrovascular Accidents[Title/Abstract]))) AND ((("Ischemia"[Mesh]) OR "Brain Ischemia"[Mesh]) OR ((((((((Ischemia[Title/Abstract]) OR (Ischemias[Title/Abstract])) OR (Brain Ischemia[Title/Abstract])) OR (Brain Ischemias[Title/Abstract])) OR (Ischemic Encephalopathy[Title/Abstract])) OR (Ischemic Encephalopathies[Title/Abstract])) OR (Cerebral Ischemia[Title/Abstract])) OR (Cerebral Ischemias[Title/Abstract])))) AND (("Tenecteplase"[Mesh]) OR (((Metalyse[Title/Abstract]) OR (TNKase[Title/Abstract])) OR (Tenecteplase[Title/Abstract])))) AND (((((((("Randomized Controlled Trial" [Publication Type]) OR "Randomized Controlled Trials as Topic"[Mesh]) OR "Random Allocation"[Mesh]) OR "Double-Blind Method"[Mesh]) OR "Single-Blind Method"[Mesh]) OR "Placebos"[Mesh]) OR ((random*[Text Word]) OR (placebo[Title/Abstract]))) OR (((((singl*[Text Word]) OR (doubl*[Text Word])) OR (trebl*[Text Word])) OR (tripl*[Text Word])) AND (((mask*[Text Word]) OR (blind*[Text Word])) OR (dumm*[Text Word]))))**
